# Impact of hiatal hernia on histological pattern of non-erosive reflux disease

**DOI:** 10.1186/1471-230X-5-2

**Published:** 2005-01-09

**Authors:** Anthie Gatopoulou, Konstantinos Mimidis, Alexandra Giatromanolaki, Vassilios Papadopoulos, Alexandros Polychronidis, Nikolaos Lyratzopoulos, Efthimios Sivridis, Georgios Minopoulos

**Affiliations:** 1 Endoscopy Unit, Democritus University of Thrace, Dragana, GR-68100 Alexandroupolis, Greece; 2 First Department of Internal Medicine, Democritus University of Thrace, Dragana, GR-68100 Alexandroupolis, Greece; 3 Department of Pathology, Democritus University of Thrace, Dragana, GR-68100 Alexandroupolis, Greece; 4 Second Department of Surgery, Democritus University of Thrace, Dragana, GR-68100 Alexandroupolis, Greece; 5 First Department of Surgery, Democritus University of Thrace, Dragana, GR-68100 Alexandroupolis, Greece

## Abstract

**Background:**

Hiatus hernia (HH) has major pathophysiological effects favoring gastroesophageal reflux and hence contributing to esophageal mucosa injury, especially in patients with severe gastroesophageal disease. However, prospective studies investigating the impact of HH on the esophageal mucosa in non-erosive reflux disease (NERD) are lacking. This study evaluated the association between the presence of (HH) and the histological findings in symptomatic patients with NERD.

**Methods:**

Fifty consecutive patients with gastroesophageal reflux disease (GERD) were enrolled. After conventional endoscopy, Lugol solution was applied and biopsy specimens were obtained. Histological parameters including basal zone hyperplasia, papillary length and cellular infiltration were evaluated. The chi-square test with Yates' correlation was used for comparing discrete parameters between groups. However, Fisher's exact probability test was used where the expected frequencies were lower than 5. Wilcoxon's test for unpaired samples was preferred in cases of semi-quantitative parameters.

**Results:**

The presence of HH along with more severe findings (0.01 <*P *< 0.05) was confirmed in 18 patients. NERD was observed in 29 (58%) patients. Basal zone hyperplasia and loss of glycogen accompanied HH in all cases, and the correlation was significant in NERD (*P *< 0.001). The remaining histological patterns were similar between erosive reflux disease and NERD in the presence of HH.

**Conclusion:**

The presence of HH is correlated with more severe endoscopy findings, and predisposes for severe histological abnormality in cases of NERD.

## Background

Gastroesophageal reflux disease (GERD) is a common condition that affects 25–30% of the population [1]. It clearly involves multifactorial pathophysiology, yet the factors underlying why only some patients develop reflux esophagitis are unclear [2].

Symptoms and demographic data do not allow differentiation between the endoscopy-negative (non-erosive reflux disease; NERD) and endoscopy-positive (erosive reflux disease; ERD) forms of the disease. In fact most patients with typical symptoms of GERD have normal esophageal mucosa on upper endoscopy. Indeed, more than two-thirds of all patients with reflux symptoms never develop esophageal erosions, ulcers or strictures [3]. This group of NERD patients constitutes a significant clinical problem since they appear to be relatively resistant to proton-pump inhibitors (PPIs) [4,5].

Hiatal hernia (HH) has been considered to be one of the pathophysiological mechanisms that contributes to the development of GERD, promoting refluxate access and impaired acid clearance; however, the impact of this mechanism in NERD is unclear [2,6,7].

The aim of the present study was to clarify the possible association of HH with histological findings on a group of prospectively studied symptomatic patients with NERD.

## Methods

Fifty patients (29 men, 21 women; aged 49.9 ± 6.6 years, mean ± SD) were evaluated prospectively in our endoscopy unit for symptoms compatible with GERD, namely heartburn, acid regurgitation and belching. A standardized questionnaire was completed for each patient during an interview with an experienced gastroenterologist. Demographic details of the GERD patients were recorded, including age, sex, smoking habits, tea, coffee and alcohol consumption, and concurrent medical conditions including hypertension and diabetes mellitus.

None of the patients included in this study had a current or past history of peptic ulcer disease, previous gastric surgery, antihelicobacter therapy, or use of PPIs, non-steroidal anti-inflammatory drugs, steroids or tetracycline during the previous 4 weeks. Ethics approval was obtained from the ethics committee of the University Hospital of Alexandroupolis, and patients provided signed, informed consent for their biopsy specimens to be taken.

Routine endoscopy was performed in all patients by the same endoscopist using a flexible endoscope (GIF-Q145, Olympus). The distance between the esophagogastric junction and the incisor teeth was recorded. Reflux esophagitis was graded in accordance with the Los Angeles classification [8]. HH was considered present if gastric folds were assessed as extending ≥2 cm above the diaphragmatic hiatus during quiet respiration [2].

At least four biopsy specimens were taken at 3 cm above the lower esophageal sphincter with biopsy forceps (Olympus) in a criss-cross manner. In order to improve endoscopic visualization and provide biopsy orientation, 20 ml of 2% potassium iodine solution (Lugol) was applied through a "spray" catheter [9-11]. To obtain sufficient material and to ensure an almost vertical pinch biopsy specimen, the opened forceps were withdrawn towards the tip of the endoscope, which was bent forwards maximally, and hence the forceps were pressed vertically against the esophageal wall. Specimens were fixed in 40 mg/L formaldehyde [12].

After all the sections had been obtained, they were assessed histologically in a blinded manner (i.e. without endoscopic or clinical information). Standardized reports completed by the histopathologist comprised an evaluation of the following histological parameters: basal zone hyperplasia, papillary length, dilatation of intraepithelial blood vessels, and semi-quantitative cellular infiltration by T-lymphocytes, neutrophils and eosinophils. Alterations in glycogen content, erosion, ulceration and chronic inflammation were also assessed as described previously [12-17].

The chi-square test with Yates' correlation was used to compare discrete parameters between groups. However, Fisher's exact probability test was used where expected frequencies were lower than 5. Wilcoxon's test for unpaired samples was preferred in cases of semi-quantitative parameters due to its greater power. Mean values and their 95% confidence limits were calculated. Statistical significance was set at *P *≤ 0.05. All analyses were performed using the statistical software package "Statistica (version 6)".

## Results

### Endoscopy findings

Endoscopy revealed esophageal mucosa with a normal appearance in 29 patients. The remaining 21 patients had esophagitis of variable severity (Table 1).

**Table 1:** Endoscopy findings in patients with reflux disease. Endoscopy findings in patients with reflux disease, for HH+ and HH-.

	NERD	ERD grade A	ERD grade B	ERD grade C	ERD grade D	Total
HH+	7	5	4	2	0	18
HH-	22	8	2	0	0	32
**Total**	29	13	6	2	0	50

**Figure 1:**
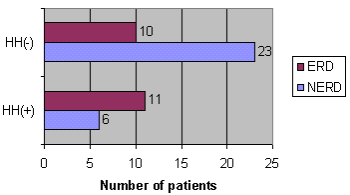
**Prevalence of HH among ERD and NERD patients. **Prevalence of HH among ERD and NERD patients. *P *= 0.0196 when HH+ and HH- are compared.

**Figure 2:**
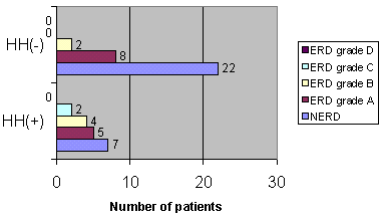
**Relationship between HH and endoscopy findings (0.01 <*P *< 0.05). **Relationship between HH and endoscopy findings. 0.01 <*P *< 0.05 when HH+ and HH- are compared.

HH was observed in 18 patients. Its presence (HH+) was correlated not only with the presence of erosions (*P *= 0.0196) (Figure 1), but also with the severity of the endoscopy findings (Wilcoxon's *T*_1 _score for unpaired samples: 576 for *N*_1 _= 18 and *N*_2 _= 32, 0.01 <*P *< 0.05) (Figure 2).

### Histological findings

Histological examinations of the biopsy specimens revealed esophagitis in 46 out of 48 patients, despite the normal appearance of the esophageal mucosa in most of them. Two specimens – one from a patient with ERD with HH and one from a patient with NERD with HH – were quantitatively inadequate and thus omitted.

**Figure 3:**
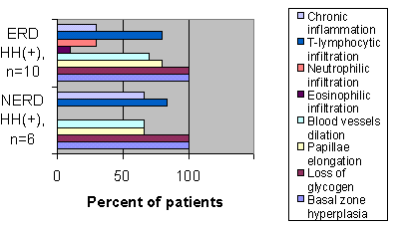
**Histological findings among ERD and NERD patients in the presence of HH. **Histological findings among ERD and NERD patients in the presence of HH. Basal zone hyperplasia and loss of glycogen is a ubiquitous histological feature in both ERD and NERD with HH. No statistically significant difference was observed between ERD and NERD with HH in any of the histological findings.

**Figure 4:**
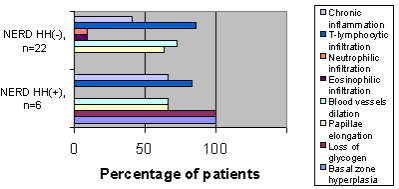
**Histological findings among NERD patients with and without hernia. **Histological findings among NERD patients with and without hernia. *P *= 2.61 × 10^-6 ^for basal zone hyperplasia and papillary elongation.

Although the remaining histological patterns were similar between ERD and NERD in HH+ (Figure 3), basal zone hyperplasia and loss of glycogen accompanied HH in all cases, with the correlation being highly significant in NERD (*P *= 2.61 × 10^-6^) (Figure 4).

## Discussion

The clinical spectrum of GERD is diverse. The disease follows a rather benign course in most patients. Indeed, it is estimated that NERD accounts for up to 70% of patients with GERD [1]. The pathophysiological mechanisms that contribute to the development of GERD include delayed gastric emptying, frequent and transient relaxation of the lower esophageal sphincter, impaired esophageal clearance of regurgitated gastric acid, and HH+ [2].

HH has recently re-emerged as an important factor in GERD [6,7,18]. It may diminish lower esophageal sphincter pressure, promote acid reflux and compromise emptying of the refluxate from the distal esophagus, prolonging acid contact with the esophageal mucosa [19-21], a mechanism that could explain the association of HH with more severe reflux [22,23]. Thus, although HH has been established as the strongest predictor of the presence and severity of esophagitis in GERD patients with esophagitis, there are no published data on the role of HH in symptomatic patients without endoscopic esophagitis.

Our prospective study suggests that HH+, even in patients with an esophageal mucosa that appears normal endoscopically (NERD), indicates the existence of histological effects.

Our population was characterized by similar clinical presentation, and HH was correlated not only with the presence of erosions (Figure 1) but also with the severity of the endoscopy findings (Figure 2). These results further support HH as a dominant predictive factor for erosive esophagitis, which has already been confirmed in previous studies [2,24-27].

In order to further investigate the role of HH in NERD patients, we studied the role of HH+ on the histological parameters of esophagitis. In our material, basal zone hyperplasia and loss of glycogen content was detected in all HH+ ERD patients and HH+ NERD patients (Figure 3). In contrast, no NERD patient without HH (HH-) exhibited similar histological abnormalities (Figure 4). These findings probably indicate that the development of NERD in HH+ patients is more closely related to the pathophysiology of ERD, and perhaps different from the mechanisms responsible for NERD in HH- patients.

Little is known about the relationship between HH and the histological variables in non-erosive esophagitis. Our finding that basal zone hyperplasia and loss of glycogen content are more frequently prevalent in HH+ than in HH- among NERD patients as well as the fact that basal zone hyperplasia, loss of glycogen content and infiltration with T-lymphocytes are more frequent in ERD than in NERD suggests the that HH contributes directly to the development of both GERD and NERD, perhaps through decreased acid clearance.

## Conclusions

HH+ not only appears to be a risk factor for NERD, but is also suggestive of the histological presence of microscopic GERD in symptomatic NERD patients. This finding could play an important role in the therapeutic management of NERD patients with PPIs in the future, since ERD patients respond better than NERD patients to antireflux therapy. Future studies should establish whether there is a cause-and-effect relationship between HH and response to PPIs in NERD patients.

## List of abbreviations

HH: Hiatal hernia

NERD: Non-erosive reflux disease

GERD: Gastroesophageal reflux disease

ERD: Erosive reflux disease

HH+: Presence of hiatal hernia

HH-: Absence of hiatal hernia

PPI: Proton-pump inhibitors

## Competing interests

The author(s) declare that they have no competing interests.

## Authors' contributions

A.G. **participated in the **endoscopy studies and in the preparation of the manuscript.

K.M. participated in the endoscopy studies and in the preparation of the manuscript.

A.G. participated in the histological studies.

V.P. contributed to the design of the study, performed the statistical analysis and produced the graphical presentations of the results.

E.S. participated in the histopathological studies.

A.P. contributed to the design of the study and critically reviewed the manuscript.

N.L. contributed to the design of the study and critically reviewed the manuscript.

G.M. coordinated the study.

All the authors read and approved the final version of the manuscript.

## Pre-publication history

The pre-publication history for this paper can be accessed here:

http://www.biomedcentral.com/1471-230X/5/2/prepub
